# GPU-accelerated meshfree computational framework for modeling the friction surfacing process

**DOI:** 10.1007/s40571-025-01048-2

**Published:** 2025-08-20

**Authors:** Ahmed Elbossily, Zina Kallien, Rupesh Chafle, Kirk A. Fraser, Mohamadreza Afrasiabi, Markus Bambach, Benjamin Klusemann

**Affiliations:** 1 Institute for Production Technology and Systems, Leuphana Universität Lüneburg, Universitätsallee 1, 21335 Lüneburg, Germany; 2 Institute of Material and Process Design, Helmholtz-Zentrum Hereon, Max-Planck-Straße 1, 21502 Geesthacht, Germany; 3 National Research Council Canada, Saguenay, QC Canada; 4 Advanced Manufacturing Lab, ETH Zurich, Zurich, Switzerland; 5 Computational Manufacturing Group, inspire AG, Zurich, Switzerland

**Keywords:** Meshless methods, Smoothed particle hydrodynamics, GPU computing, Friction surfacing

## Abstract

**Abstract:**

This study presents a meshfree framework for modeling the friction surfacing (FS) process using the smoothed particle hydrodynamics (SPH) method. The framework leverages GPU computing to address the computational demands of SPH, incorporates optimization techniques such as particle switching and sub-domain division to enhance simulation time efficiency, and integrates artificial viscosity, artificial stress, and kernel correction for simulation stability. A novel criterion for material separation based on joining temperature and critical shear stress is proposed for the rod material, providing accurate results in terms of the deposited material to the substrate during FS. Furthermore, the model is successfully validated to experimental observations of FS of the aluminum alloy AA5083 in terms of axial force, temperature profiles, and deposit geometries, proving the main dependencies of process parameters on deposit width and thickness. The SPH model provides in-depth insight into the deposition mechanisms, particularly illustrated in terms of material flow, deposited material distribution, and rod flash formation, aligning well with experimental findings. The simulations confirm the deposit shift toward the advancing side, where the maximum temperature is also observed. High plastic strain is concentrated in the rod flash and deposit, with higher values on the advancing side than the retreating side. The validated 3D SPH model provides a robust tool for predicting the thermo-mechanical behavior in FS processes, offering insights to advance the understanding and optimization of this deposition technique.

**Graphic Abstract:**

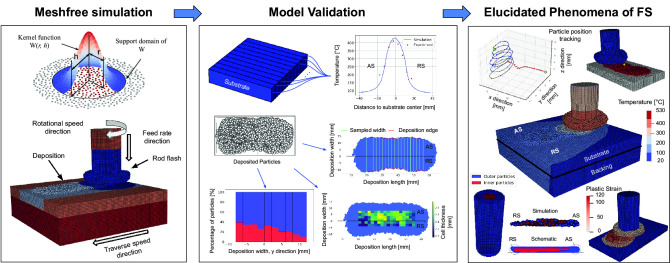

## Introduction

Friction surfacing (FS) is a solid-state coating process that enables the deposition of a consumable material via frictional heat and plastic deformation. In this process, a rotating consumable rod is pressed onto the substrate surface under an applied axial load. As the rotating rod rubs against the substrate, the softened material in this zone experiences shear deformation. This shearing action essentially breaks down the material structure, and a layer of material is deposited on the substrate. The deposited layer consolidates and forms a new interface to the rotating rod. A continuous deposited material layer is obtained by applying a traverse speed to the substrate, and a mushroom-shaped flash of material is formed at the rod tip along the deposition process [1]. FS has a lower heat input compared to fusion-based processes due to its solid-state nature, where the maximum temperature does not reach the material’s melting temperature. This minimizes the size of the heat-affected zone (HAZ) and prevents distortion of larger parts [2]. Moreover, dynamic recrystallization leads to the formation of a fine-grained microstructure of the deposit due to the thermo-mechanical input during the process [1]. Due to these advantageous features, FS represents a prominent technology for coating and repair applications [3]. Recently, it also gained attention in terms of additive manufacturing [4–6].

Several experimental studies have investigated the FS process, for instance, in terms of the effect of the process parameters on the process characteristics. Fitseva et al. [7] indicated that higher rod rotational speeds combined with increased axial forces result in more efficient deposition. Rahmati et al. [8] revealed that an increase in the rotating velocity of the consumable rod necessitates a reduction in either the axial feed rate or the traverse velocity. Rafi et al. [9] found that slower rod rotation creates wider coatings, where faster traverse speed results in thinner coatings. Kallien et al. [10] showed that axial force, rotational speed, backing material, and substrate thickness affect process temperatures, which in turn determine the final deposit geometries.

Although there exists a significant number of experimental studies investigating the FS process, there remain significant challenges in terms of process understanding. The continuous consumption of the rod and intense forces on the substrate surface make temperature measurements as well as material flow investigations difficult, especially at the critical rod–substrate contact zone. This necessitates the use of numerical methods to investigate the FS process. Initial numerical models often employed the finite element method (FEM), which primarily focused on the temperature field [11, 12]. In these models, the deposited material was typically introduced using the element activation technique (e.g., [12]), thus neglecting the actual material flow. Next to purely thermal analysis, studies on the thermo-mechanical response were also performed via FEM in a similar fashion [13, 14]. In general, mesh-based discretization techniques such as the FEM encounter difficulties when dealing with material separation and severe plastic deformation, both present in the FS process. As an alternative, meshless approaches are promising, where the smoothed particle hydrodynamics (SPH) approach presents one of the prominent methods [15].

The general idea of SPH is to approximate a continuous field using a set of kernel functions centered at discrete particles, which carry the physical properties of the system. SPH has been successfully used to model solid-state processes, such as friction stir welding (FSW), additive friction stir deposition (AFSD), and friction extrusion (FE). For instance, Tartakovsky et al. [16] used SPH for simulating the FSW process, but the model dimensions were limited due to high computational costs. To address the high computational costs, Fraser et al. [17] presented a GPU-based SPH framework for simulating FSW, enabling efficient computation of temperature, stress, and material flow. Using this framework, Stubblefield et al. [18] developed a model to simulate AFSD, which captured the main process characteristics, such as the temperature differences between the advancing side (AS) and retreating side (RS). Palya et al. [19] used SPH to predict the performance of AFSD repairing, which was integrated into a fatigue model to estimate fatigue life performance. Li et al. [20, 21] presented the successful application of SPH to model FE in terms of material flow. In a subsequent study, Li et al. [22] incorporated a constitutive model based on dislocation density in the SPH framework, leading to significant improvements in the prediction of material behavior at larger deformation.

Although meshfree simulations have been successful in simulating many solid-state processes, their use for FS is still scarce, in particular due to the underlying separation process. While Aval et al. [23–25] applied SPH to FS, their model did not cover in-depth aspects of material transfer from the rod to the substrate. Furthermore, their study was based on a CPU implementation, leading to certain restrictions in terms of resolution due to the simulation time.

These aspects are addressed in this work. In this regard, a fast, robust, and comprehensive framework on the basis of SPH is developed for the FS process. The framework is based on a GPU-based framework [26], implemented using C++/CUDA [27]. A meshfree SPH approach is employed with a combination of stabilization terms to achieve a robust and accurate simulation of the FS process. A novel joining criterion based on joining temperature and critical shear stress is proposed, allowing to accurately model the transition from rod to deposited material during FS. The simulations are validated against experimental data, showing their accuracy in capturing the key thermo-mechanical phenomena. Simulation results enable detailed tracking of material transformation from the rod to the substrate and prediction of rod material distribution within the deposit, contributing to a deeper understanding of FS and its optimization.

The paper is organized as follows: Sect. 2 details the experimental procedure used for FS. Section 3 discusses the FS model formulation in a meshfree updated Lagrangian framework. Section 4 outlines the model setup. Section 5 presents the numerical results, validates them against experimental data, and discusses the obtained results. Finally, Sect. 6 concludes the paper with remarks.

**Table 1:** Overview of process parameters and resulting average feed rate for experimental FS layer depositions obtained in [10]

Set	Axial force	Rotational speed	Traverse speed	Feed rate
	(kN)	(rpm)	(mm/s)	(mm/s)
A	8	1200	6	1.83
B	8	900	6	1.86
C	8	1500	6	1.83
D	8	1200	4	1.93
E	8	1200	8	2.18

**Fig. 1:**
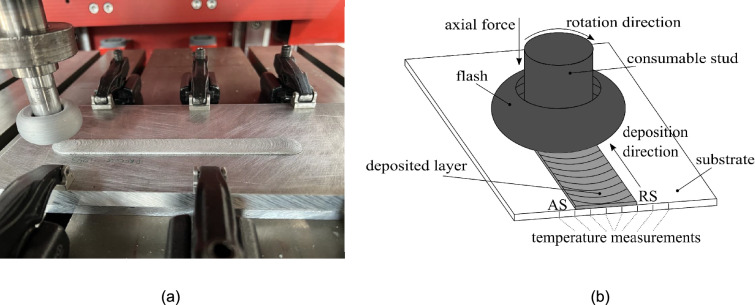
Solid-state layer deposition via FS, showing **a** the experimental deposition of one single layer and **b** a schematic of the FS process, where the main process parameters are axial force, rotational, and traverse speed

## Experimental setup

The FS experimental data taken for the validation of the model is mainly based on a previous experimental study [10]. The materials for the experimental part of this work were the aluminum alloy AA5083-H112 as consumable studs (125 mm length, 20 mm diameter) and the aluminum alloy AA7050-T7451 as substrates (300 mm length, 130 mm width, 10 mm thickness). An AA7050-T84 backing plate (300 mm length, 130 mm width, 8 mm thickness) was used between the substrate and machine table. All experimental FS layer depositions were performed at room temperature using special-purpose friction welding equipment (RAS, Henry Loitz Robotik, Germany), where the three main process parameters are axial force, rotational speed, and traverse speed. The FS process begins with the dwelling phase, where the rotating rod is pressed onto the substrate at a defined force without applying any traverse speed. Following this, the process shifts to the deposition phase, during which a constant traverse speed is applied to the substrate. The corresponding FS experiments were performed in force control, i.e., the resulting rod feed rate is an output parameter that is calculated from the axial displacement and time recorded by the welding equipment throughout the layer deposition. During the deposition phase, the process presents a stable behavior in terms of torque, force, and displacements. The process can be considered to be in a steady state after approximately 35 mm of deposition. The feed rate was calculated over the next 100 mm of deposition. An overview of the considered FS layer depositions is given in Table 1. The feed rate depends on the axial force as well as the process temperature [10]. For a variation in rotational speed for sets A and B, while keeping the axial force constant, the change in process temperature was not very significant, which explains the similar values in feed rate. The process temperatures were recorded at 50 Hz using thermocouples (Type K) positioned 0.5 mm below the substrate’s surface at different distances from the considered center position of the deposit. Figure 1a shows the experimental FS process, while Fig. 1b provides a schematic overview. The thickness and width of the deposits were determined from cross sections taken from the FS deposited layers. For more details on the experimental procedures, the interested reader is referred to [10].

## Meshfree formalism

This section covers the discretization of the FS model’s governing equations using the SPH method and the applied contact and friction forces. Moreover, it discusses the material model and heat generation. For completeness, details on the notation used in this work are provided in “Appendix A”.

**Fig. 2:**
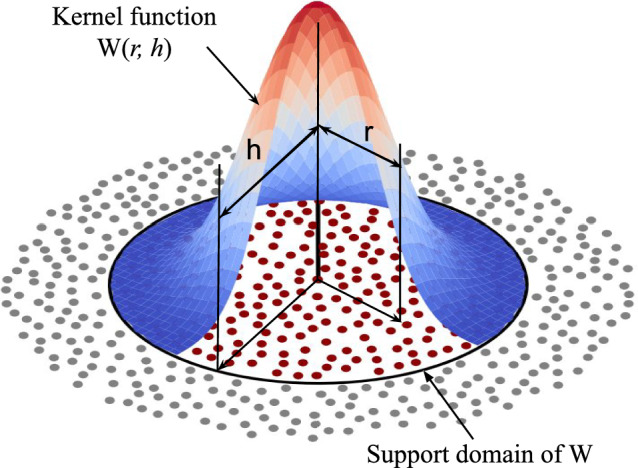
Schematic of kernel function interpolation in SPH method (for 2D)

### Discretized governing equations

The governing system for solid mechanics can be expressed using three conservation laws: mass, momentum, and energy. Additionally, there are a constitutive equation and a kinematic equation to be solved. The SPH method solves the field equations using interpolation with a kernel function, *W*(*r*, *h*) [28]. The kernel function operates on a set of neighboring material particles that are within a smoothing length *h* of the particle of interest, see Fig. 2. The smoothing length defines a cutoff radius around the particle of interest. The smoothing length is determined via the product of the smoothing length factor $h_f$ and initial particle spacing $\Delta x$, where the smoothing length factor represents a user-defined parameter. By using kernel interpolation, the SPH method can accurately approximate the field variables and their derivatives, ensuring smooth and continuous solutions without the need for a predefined mesh structure. The system of governing equations is approximated using the SPH method as

1 $$\begin{aligned}  &   {\dot{\rho }}_{i}=-\rho _{i} \sum _{j=1}^{N} V_{j}\left[ {\varvec{v}}_{j}-{\varvec{v}}_{i}\right] \cdot \varvec{ \nabla } W_{ij}, \end{aligned}$$

2 $$\begin{aligned}  &   \dot{{\varvec{v}}}_{i}=\sum _{j=1}^{N} m_{j}[\frac{\varvec{\sigma }_{i}}{\rho _{i}^{2}}+\frac{\varvec{\sigma }_{j}}{\rho _{j}^{2}}+\underbrace{\Pi _{ij} {\varvec{I}}+{\varvec{R}}_{ij}}_{stabilizers }] \varvec{ \nabla } W_{ij}+\frac{1}{m_{i}} {\varvec{b}}_{i}, \nonumber \\ \end{aligned}$$

3 $$\begin{aligned}  &   {\dot{T}}_{i}= \frac{1}{c_{pi} \ \rho _i} \left[ \sum _{j=1}^{N} V_{j} \frac{2k_{i}k{j}}{k_{i}+k{j}} \left[ T_{j}-T_{i}\right] \nabla W_{ij}^{\text {PSE}} \right. \nonumber \\  &   \qquad \quad \left. + {\dot{q}}_i^{fric} + {\dot{q}}_i^{pl} + {\dot{q}}_i^{diss}\right] , \end{aligned}$$

4 $$\begin{aligned}  &   \dot{\varvec{{x}}}_{i}={\varvec{v}}_{i}+ \underbrace{\beta \sum _{j=1}^{N} \frac{m_{j}}{\rho _{i}+\rho _{j}}\left[ {\varvec{v}}_{j}-{\varvec{v}}_{i}\right] W_{i j}}_{\textrm{Xsph} \text{ correction } }, \end{aligned}$$

where $\rho _i$, ${\varvec{v}}_i$, $T_i$, and ${\varvec{x}}_i$ are the density, velocity, temperature, and position of particle *i*, respectively. $V_{j}=m_{j} / \rho _{j}$ is the volume represented by particle *j*. ${\varvec{\sigma }}_{i}$ and ${\varvec{\sigma }}_{j}$ are the stress tensors of particle *i* and *j*, respectively. ${\varvec{I}}$ is the identity tensor. $\Pi _{ij}$ is called the artificial viscosity and ${\varvec{R}}_{ij}$ artificial stress, see “Appendix B”. ${\varvec{b}}_{i}$ are the body forces, and $c_{pi}$ and $k_i$ are the heat capacity and the thermal conductivity, respectively. The harmonic mean of the thermal conductivities $\frac{2k_{i}k{j}}{k_{i}+k{j}}$ in Eq. (3) is used for enhancing the stability [29]. ${q}_i^{fric}$ and ${q}_i^{pl}$ are the heat generated due to friction and plastic deformation, respectively. ${q}_i^{diss}$ is the dissipated heat to the surroundings. $\beta$ is the Xsph modified velocity factor, which is used to prevent the particles from clustering [30]. The “Cubic B-spline” smoothing function [31] employed as smoothing kernel $W_{ij}$ is given as

5 $$\begin{aligned} W_{ij}=n_{c} {\left\{ \begin{array}{ll} 1-\frac{3}{2} R^{2}+\frac{3}{4} R^{3}, &  0 \le R<1 \\ \frac{1}{4}[2-R]^{3}, &  1 \le R \le 2 \\ 0, &  \text {otherwise} \end{array}\right. } \end{aligned}$$

where the normalization factor $n_{c}=\frac{1}{\pi h^{3}}$ (for 3D) and $R=\frac{\Vert {\varvec{x}}_{i j}\Vert }{h}$ are used. ${\varvec{x}}_{ij}$ is the distance between the two particles *i* and *j*. In this regard, the gradient of the smoothing kernel reads as follows

6 $$\begin{aligned} \varvec{\nabla } W_{ij}=\frac{{\varvec{x}}_{i j}}{\Vert {\varvec{x}}_{i j}\Vert } \frac{\partial W_{i j}}{\partial R}. \end{aligned}$$

Particle strength exchange (PSE) kernel is used for the second derivative approximation in Eq. (3) as recommended by Afrasiabi et al. [26, 32]. $\nabla W_{ij}^{\text {PSE}}$ is given by

7 $$\begin{aligned} \nabla W_{ij}^{\text {PSE}}=\frac{4}{\pi ^\frac{3}{2} h^5} \sum _{j=1}^{N} e^{-R^2}. \end{aligned}$$

The conventional SPH method, as presented in Eqs. (1)–(4), has a limitation in reconstructing even a linear function throughout the entire domain [33, 34] due to a boundary deficiency. This limitation is a well-known drawback in meshfree simulations. To address this shortcoming, the corrected smoothed particle method (CSPM) [35] is used for the calculation of the spatial gradient of any arbitrary function $f({\varvec{x}}_i)$, which is expressed

8 $$\begin{aligned}  &   {{\varvec{\nabla }} f\left( {\varvec{x}}_{i}\right) ^{\textrm{CSPM}}}={\varvec{A}}_{i}^{-1}\left[ \sum _{j=1}^{N} V_{j}\left( f\left( {\varvec{x}}_{j}\right) -f\left( {\varvec{x}}_{i}\right) \right) \varvec{\nabla } W_{ij}\right] ,\nonumber \\ \end{aligned}$$

9 $$\begin{aligned}  &   {\varvec{A}}_{i}=\sum _{j=1}^{N} V_{j}\left( {\varvec{x}}_{ji} \otimes \varvec{\nabla } W_{i j}\right) . \end{aligned}$$

${\varvec{A}}_{i}$ is the second-order CSPM re-normalization tensor for the first derivatives of particle *i*. FS simulations require CSPM as high-speed particles on the surface of the rod suffer from this well-known boundary deficiency. Omitting CSPM leads to irregular particles distribution on the rod outer surface and eventually the failure of the numerical simulation. Particles on the rod outer surface have only half, or less, of the usual kernel support. CSPM effectively re-centers and rescales the kernel gradient so that—even at a half-support boundary—it reproduces linear fields. That eliminates the imbalances in neighbors, preserves uniform spacing, and prevents the particles from clustering and void formation on the rod outer surface, see “Appendix C”.

### Contact and friction forces

The contact force is calculated based on the notion that each particle possesses a spherical volume that is impenetrable by any other body particle [36]. To determine whether a body particle is penetrating into another body particle, the distance between them is calculated and compared with the initial discretization distance. Then, the penalty contact force is given by

10 $$\begin{aligned} {\varvec{f}}_{i}^{{con}}=\sum _{j \in M_{i}^{B_{c}}}[\lambda _{1} {\dot{p}}_{ij}^{n_{{av}}}+\lambda _{2} p_{ij}^{n_{{av}}}] A V_{j} W_{ij} {\varvec{n}}_{ij}^{{av}}, \end{aligned}$$

where $M_{i}^{B_c}$ is the set of neighboring contact particles for particle *i*, determined by the condition $||x_{ij}|| < \Delta x$. $A = \pi \Delta x h$ and $p_{ij}^{n_{{av}}}$ is the average penetration distance, ${\dot{p}}_{ij}^{n_{{av}}}$ is the average penetration rate, and ${\varvec{n}}_{ij}^{{av}}$ is the average normal vector between the two contact particles, see “Appendix D”. $\lambda _{1}$ and $\lambda _{2}$ are calculated as

11 $$\begin{aligned} \lambda _{1}=\frac{\rho _{j} c_{j}}{\rho _{j} c_{j}+\rho _{i} c_{i}} \rho _{i} c_{i}, \end{aligned}$$

12 $$\begin{aligned} \lambda _{2}=\frac{E_{i} E_{j}}{E_{i}+E_{j}} \frac{1}{\Delta x}, \end{aligned}$$

where $E_{i}$ and $E_{j}$ are the modulus of elasticity of the particles in contact. The penetration distance $p_{ij}^{n_{{av}}}$ and the penetration rate ${\dot{p}}_{ij}^{n_{{av}}}$ are given by

13 $$\begin{aligned}  &   p_{ij}^{n_{{av}}}=\left[ \Delta x -|{\varvec{{x}}}_{ij} \cdot {\varvec{{n}}}_{ij}^{av}|\right] , \end{aligned}$$

14 $$\begin{aligned}  &   {\dot{p}}_{ij}^{n_{{av}}}=\left[ {\varvec{{v}}}_{i} - {\varvec{{v}}}_{j}\right] \cdot {\varvec{{n}}}_{ij}^{av}. \end{aligned}$$

The friction force is calculated based on the classical Coulomb friction model using a constant coefficient of friction $\mu$. The friction force acts on the contact tangential plane and is calculated as follows

15 $$\begin{aligned} {\varvec{{f}}}_{i}^{fric}=\mu \left\| {\varvec{f}}_i^{{con}}\right\| \frac{{\varvec{{v}}}_{ij}^{rel,t}}{\left\| {\varvec{{v}}}_{ij}^{rel,t}\right\| }. \end{aligned}$$

${{\varvec{v}}}_{ij}^{rel,t}$ denotes the relative tangential velocity, given as

16 $$\begin{aligned} {\varvec{{v}}}_{ij}^{rel,t}= {\varvec{{v}}}_{ij}^{rel} - {\varvec{{v}}}_{ij}^{rel} \circ {\varvec{{n}}}_{ij}^{av}, \end{aligned}$$

where ${{\varvec{v}}}_{ij}^{rel}$ denotes the relative velocity, determined via

17 $$\begin{aligned} {\varvec{{v}}}_{ij}^{rel}= {\varvec{{v}}}_{i} - {\varvec{{v}}}_{j}. \end{aligned}$$

### Material model

Hooke’s law is used for modeling the linear elastic material behavior. Jaumann rate [37] is employed for modeling the deviatoric stress ${\varvec{S}}$ instead of the rate of the Cauchy stress because of its lack of frame invariance. By assuming an isotropic material, the rate of change of the deviatoric stress ${\varvec{{\dot{S}}}}$ is given by

18 $$\begin{aligned} \varvec{{\dot{S}}}= \textrm{2G} [\varvec{{\dot{\varepsilon }}} - \frac{\textrm{tr}(\varvec{{\dot{\varepsilon }}})}{3} {\textbf {I}}]+ \varvec{\Omega } \cdot {\textbf {S}} - {\textbf {S}} \cdot \varvec{\Omega }. \end{aligned}$$

G is the shear modulus and **I** is the identity matrix. $\varvec{{\dot{\varepsilon }}}$ is the strain rate tensor, and $\varvec{\Omega }$ is the skew-symmetric parts of the velocity, given as

19 $$\begin{aligned} \varvec{{\dot{\varepsilon }}} = \frac{1}{2}[\nabla {\varvec{{v}}} + (\nabla {\varvec{{v}}})^T], \end{aligned}$$

20 $$\begin{aligned} \varvec{\Omega } = \frac{1}{2}[\nabla {\varvec{{v}}} - (\nabla {\varvec{{v}}})^T]. \end{aligned}$$

The total stress $\varvec{\sigma }$ is defined as the subtraction of the hydrostatic stress ${\textbf {S}}_h$ from the deviatoric one ${\textbf {S}}$ [38], expressed as

21 $$\begin{aligned}  &   \varvec{\sigma } = {\textbf {S}} - {\textbf {S}}_h, \end{aligned}$$

22 $$\begin{aligned}  &   {\textbf {S}}_h = p {\textbf {I}}. \end{aligned}$$

*p* is the hydrostatic pressure, which is calculated based on the following equation according to Libersky et al. [39], who applied it to SPH:

23 $$\begin{aligned} p = c_0^2 \ [\rho - \rho _0]. \end{aligned}$$

$c_0$ and $\rho _0$ are the speed of the sound wave in the material and its initial density, respectively. The material is assumed to follow J2 plasticity theory, i.e., the plastic behavior of the material is only influenced by the deviatoric stress. $J_2$, represented by the second invariant of the deviatoric stress tensor, is calculated as

24 $$\begin{aligned} J_{2} = \frac{1}{2}[{\varvec{S}}: {\varvec{S}}]. \end{aligned}$$

This leads to a yield function given as

25 $$\begin{aligned} F(J_{2}) = \sqrt{3 J_{2}} - \sigma _y({\varepsilon }_{pl}, \dot{{\varepsilon }}_{pl}, T) = 0. \end{aligned}$$

The Johnson–Cook model [40] is employed to capture the material’s constitutive behavior, which is expressed as

26 $$\begin{aligned} \sigma _{y}\left( {\varepsilon }_{pl},\dot{{\varepsilon }}_{pl}, T\right)= &   \left[ A+B\left( {\varepsilon }_{pl}\right) ^{n}\right] \left[ 1+C \ln \left( \frac{\dot{{\varepsilon }}_{pl}}{\dot{{\varepsilon }}_{pl}^{0}}\right) \right] \nonumber \\  &   \ \left[ 1-\left( \frac{T-T_{0}}{T_{m}-T_{0}}\right) ^{m}\right] , \end{aligned}$$

where *A*, *B*, *C*, *n*, and *m* are the Johnson–Cook material parameters, determining the strain hardening, strain rate strengthening, and thermal softening behavior of the material. ${\varepsilon }_{pl}$ and $\dot{{\varepsilon }}_{pl}$ denote the equivalent plastic strain and equivalent plastic strain rate, respectively. ${\dot{\varepsilon }}_{pl}^{0}$ is the equivalent plastic reference strain rate used in determining *A*, *B*, and *n*. *T*, $T_{0}$, and $T_{m}$ are current, reference, and melting temperature, respectively.

### Heat generation and dissipation

The model incorporates two heat sources: heat generated by plasticity ${\dot{q}}_{pl}$ and heat generated by friction ${\dot{q}}_{fric}$. The ${\dot{q}}_{pl}$ is calculated as [20, 26]

27 $$\begin{aligned} {\dot{q}}_{pl}= \alpha \ {\dot{\varepsilon }}_{pl} \ \sigma _{y}\left( {\varepsilon }_{pl}, \dot{{\varepsilon }}_{pl}, T\right) , \end{aligned}$$

where $\alpha$ is the Taylor–Quinney coefficient. The other heat source ${\dot{q}}_{fric}$ is given by [20, 26]

28 $$\begin{aligned} {\dot{q}}_{fric} = {t_s} \ \left\| {\varvec{f}}_{fric} \right\| \ \left\| {\varvec{v}}_{rel,t} \right\| , \end{aligned}$$

where $t_s$ represents the heat splitting factor due to friction between the rod and the substrate. ${\varvec{f}}_{fric}$ and ${\varvec{v}}_{rel,t}$ are the friction force and the relative tangential velocity between two different bodies in contact, respectively.

The heat is dissipated to the surroundings by convection given by [41]

29 $$\begin{aligned} {\dot{q}}_{diss} = h_{c} \ A_s \ (T_{\text {ref}} -T_s ). \end{aligned}$$

where $h_{c}$ is the heat convection coefficient. $T_s$ is the particle temperature exposed to the surrounding environment, $T_{ref}$ is the surrounding reference room temperature, and the particle surface area is defined as $A_s= \Delta x^2$.

## Model setup

**Fig. 3:**
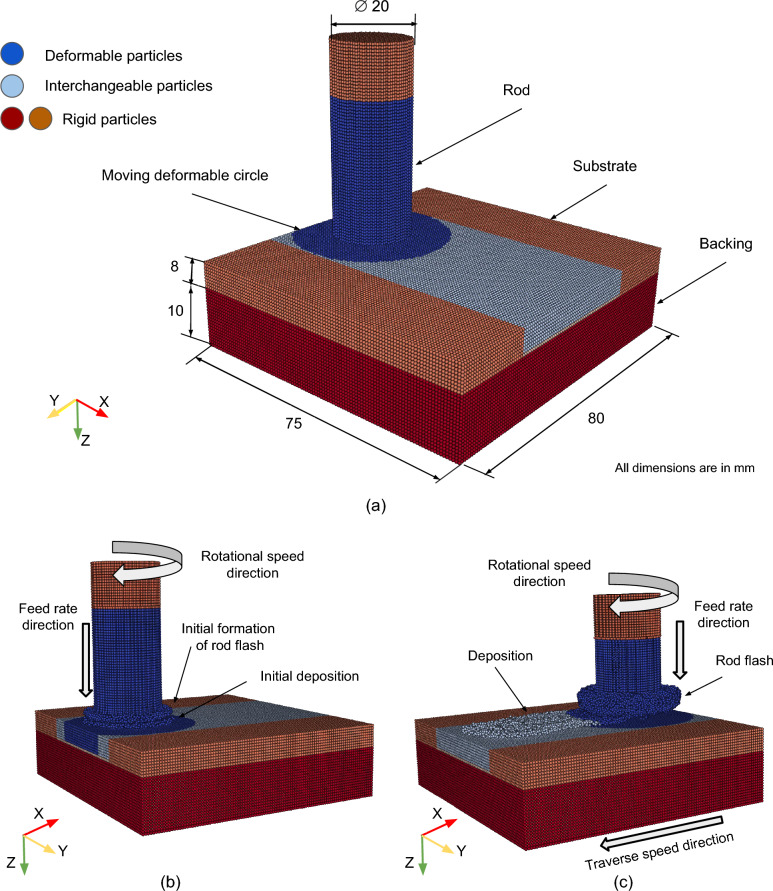
FS model used in this study: **a** the main dimensions and particle types; and **b** and **c** visualization of feed rate direction, rod rotational speed direction, and the formation of rod flash and material deposition at the end of dwelling phase and the deposition phase, respectively

The model setup for FS, depicted in Fig. 3a, consists of three key components: a deformable rotating rod representing the consumable material, a deformable substrate, and a rigid backing plate. During the simulated dwelling phase, the rod is allowed to rotate at a fixed position while being fed downward at a constant rate for a depth of 1 mm, as illustrated in Fig. 3b. Subsequently, the simulation transitions to the deposition phase, where the substrate itself begins to move laterally at a constant traverse speed and the deposit is formed over the substrate as shown in Fig. 3c.

### Model geometry and main simulation parameters

In the model, the AA5083 rod has a diameter of 20 mm and a length of 50 mm. The AA7050 substrate measures $75 \times 80 \times 8~ \text {mm}^3$. The backing plate has dimensions of $75\times 80\times 10\ \text {mm}^3$. Heat dissipates to the surroundings by convection through the outer surfaces of the model. A higher heat convection coefficient is employed on the backing plate bottom surface to mimic heat dissipation to the welding machine table [42]. The employed material parameters used in this study are given in Tables 2 and 3, where the thermo-elastic properties are used as temperature-independent. Further constants used in the simulation are summarized in Table 4. The rod is discretized initially with 35,945 particles, where 316,332 particles are used for the substrate and backing plate combined. The initial SPH particles are spaced 0.75 mm apart. The initial particle spacing is selected to balance model prediction accuracy with simulation run time. Reducing the particle size by 0.1 mm increases the simulation time by approximately 70%, with no significant improvement in prediction accuracy. Different particle types have been used (deformable, rigid), which is addressed in detail in the following sections. The developed model is used to simulate each set of process parameters listed in Table 1. To ensure numerical stability, all simulations employed displacement boundary conditions. The axial force obtained from the model is then compared against the force applied by the welding machine for model validation.

### Joining criterion

In this study, the material flow from the rod to the deposit is defined based on the particle energy and maximum allowable shear stress. For the particle to leave the rod and join the deposit, on the one hand, the particle’s energy state has to surpass a predefined level. On the other hand, the applied stress at the interface during deformation has to be sufficiently high to shear the material off, limited by the temperature-dependent shear strength of the material. The total joining criterion is defined as

30 $$\begin{aligned} \text {Joined}_{i} = \left\{ \begin{array}{ll} 1, &  (T_{i} \ge \eta T_{m}) \wedge (\varepsilon _{i}^{pl} \ge 0.01) \wedge (\tau _i \ge \tau _i^{allow}) \\ 0, &  \text {Otherwise} \end{array} \right. \nonumber \\ \end{aligned}$$

**Table 2:** Johnson–Cook material parameters of the rod and substrate materials: AA5083 [43] and AA7050 [44], respectively. No strain hardening has been assumed for the deposited material AA5083, where the influence of the strain hardening coefficient B is studied in the results section

Unit	*A* (MPa)	*B* (MPa)	*C* (−)	*m* (−)	*n* (−)	$T_{0} (^{\circ }$C)	$T_{m} (^{\circ }$C)
AA5083	167.0	0.0	0.001	0.859	0.551	20	620
AA7050	450.821	108.537	0.027	0.981	0.045	20	630

**Table 3:** Thermal properties of the rod and substrate materials: AA5083 [43] and AA7050 [44], respectively

	$\rho$	*E*	$\nu$	*k*	$c_p$
Unit	$(\mathrm {kg/m^{3}})$	$(\textrm{GPa})$	$(\mathrm {-})$	$(\mathrm {W/m\ ^{\circ }C})$	$(\mathrm {J/kg\ ^{\circ }C})$
AA5083	2700	70	0.3	117	910
AA7050	2830	70.3	0.33	167	896

where $\varepsilon _{i}^{pl}$ denotes the current plastic strain of the particle and $\eta T_m$ represents a fraction of the melting temperature. $\tau _i^{allow}$ is the maximum allowable shear stress for the particle calculated as $\frac{\sigma _i^y}{\sqrt{3}}$. This criterion essentially ensures that particles only join the deposit if they are sufficiently hot ($T_{i} \ge \eta T_{m}$) and have undergone plastic deformation ($\varepsilon _{i}^{pl} \ge 0.01$).^1^ Additionally, the novel criterion $(\tau _i \ge \tau _i^{allow})$ captures the shear-off process occurring between the rod material and the joining interface. By integrating this shear stress threshold, the joining criterion imposes a realistic physical constraint on the deposition process. This ensures that particles are mechanically separated only when sufficient shear force is applied, thereby improving deposition quality. Finally, to explore how joining temperature affects material deposition, simulations were performed for different values of $\eta$. The outcomes of these simulations will be discussed in the results section.

**Table 4:** Simulation parameters employed in the FS simulations

Parameters	Symbols	Values	Reference	Equation
Xsph	$\beta$	0.01	[18]	(4)
Smoothing length factor	$h_f$	1.7	–	(5)
Coefficient of friction	$\mu$	0.35	[32]	(15)
Taylor–Quinney coefficient	$\alpha$	0.9	[32]	(27)
Heat split factor	$t_s$	0.5	–	(28)
Heat convection coefficient	$h_{c}$	25, 1000 $[\hbox {W/m}^2 {^\circ }\textrm{C}]^\textrm{a}$	[42]	(29)
Monaghan viscosity constants	$\alpha _{\Pi }$, $\beta _{\Pi }$, $\varphi$	1.0, 1.0, $0.01h^{2}$	[45]	(B.1–B.2)
Artificial stress constants	$\epsilon$, $\gamma$	0.3, 4.0	[46]	(B.4–B.6)
Critical angle	$\theta _{c}$	$70^{\circ }$	[36]	( D.5)

$^\textrm{a}$A heat convection coefficient of $h_c = 1000 \ [\hbox {W/m}^2 {^\circ }C]$ is employed on the backing plate bottom surface, while $h_c = 25 \ [W/m^2 {^\circ }C]$ is used for all other outer surfaces of the model

### GPU implementation and enhancements

The maximum time step $\Delta t^{max}$ calculated based on the Courant–Friedrich–Lewy (CFL) condition [47] is 4.1e$-$8 s for the current model setup, which would require roughly 180 million time steps for a simulation time of 7.4 s, representing a deposited length of roughly 65 mm, depending on the specific welding parameters. Due to the high computational cost, simulating this process on the CPU with a total of 352,277 particles represents not an option. However, preliminary runs even on a single NVIDIA A100 GPU card took more than 1400 h to be completed. This time is reduced to around 50 h by introducing three key enhancements: using a velocity scale factor ($V_{sf}$), particle switching, and introducing sub-domains.

#### Velocity scale factor

This study adopts the velocity scaling technique previously utilized by Li et al. [20] for simulating the friction extrusion process. This method reduces computation time by scaling all velocities in the simulation by a factor, $V_{sf}$. However, this would lead to increased heat generation and stresses. To mitigate these effects, Fraser [48] proposed multiplying the thermal conductivity and heat convection coefficients by $V_{sf}$ and dividing the plastic strain rate by $V_{sf}$. In this study, $V_{sf}$ was set to 10, maintaining the ratio of kinetic energy to internal energy below 1.2% throughout the simulation. This approach accelerated the simulation by a factor of 10 while maintaining the physical relationships.

#### Particle switching

Figure 3 illustrates already the idea of particle switching. The bottom part of the rod (depicted in blue) is considered deformable. The upper part of the rod (depicted in orange) and backing (depicted in red) act as rigid bodies, where only the heat equation is solved. The substrate is divided into four regions: two rigid regions (depicted in orange), one deformable region (depicted in blue), and an interchangeable region (depicted in light blue). Within the blue circle and down to the substrate bottom, particles are considered deformable, while the remaining particles are treated as rigid and used for heat transfer purposes only. This contact circle is constantly updated to remain centered on the moving rod. Once a particle enters this circle region, it is switched to a deformable particle, and once it leaves, it is switched back to a rigid particle. This approach avoids solving all equations for all particles, limiting the calculation of the mechanical boundary value problem to the core of the model setup, where deformation and stresses are expected. It also eliminates the need for algorithms that detect free surfaces and contact forces between these rigid particles. This simplification leads to a 50% reduction in simulation time.

#### Using sub-domains

To determine particle interactions, each particle must identify its neighbors within a smoothing length $h$. The simplest method involves iterating through all particles in the domain to check if each one is a neighbor. This naive approach is usually improved by using a hashing algorithm [49], which divides the space into fixed-size bins with a side length $l > h$. A particle within a specific bin can then interact with other particles in the same bin and the 26 neighboring bins. However, this method requires sorting the particles based on a hash function at every time step.

**Fig. 4:**
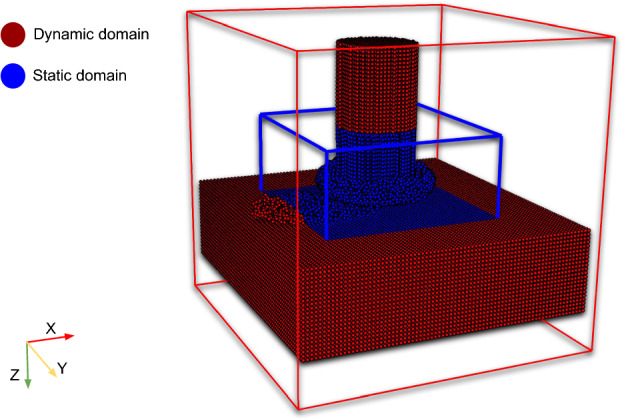
Splitting the model into sub-domains: a dynamic domain (blue) where particles undergo dynamic interactions, i.e., the critical small time step is used, and a static domain (red) where a much larger time step is applied

To minimize unnecessary sorting, the problem domain is divided into two sub-domains as illustrated in Fig. 4. The dynamic domain, where particles interact thermo-mechanically and all governing equations must be solved, is assigned a small time step, i.e., 4.1e$-$8 s, ensuring the CFL condition. Conversely, in the static sub-domain, where only the heat equation is solved, a larger time step is used of approximately 7.4e$-$05 s. This method reduces the sorting process and decreases the total running time by an additional 30%.

The optimizations introduced, including velocity scaling, particle switching, and different time steps in different sub-domains, significantly reduced the simulation running time to approximately 50 h when executed on a single NVIDIA A100 GPU card. This reduction in time demonstrates the effectiveness of the optimizations in leveraging the A100’s high-performance computing capabilities, ensuring that complex simulations can be run more efficiently while maintaining accuracy and stability.

## Results and discussion

At first, the model behavior is investigated in terms of the influence of two critical model parameters: strain hardening of the rod material and the joining temperature. Afterward, the SPH model is validated using experimental results at different process parameters, where the validation focuses on the axial force, the substrate temperature profile, deposit dimensions (width and thickness), and the resulting deposit profile. Finally, the model is used to investigate characteristic features of the FS process, including material flow and others, gaining a deeper physical understanding of the process. Details on the post-processing of the obtained simulation data are provided in “Appendix E”.

**Fig. 5:**
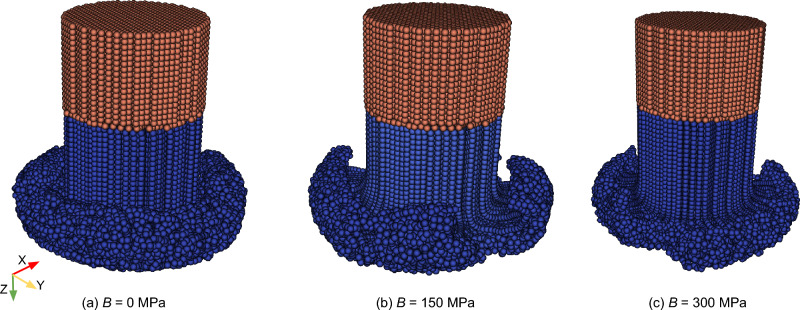
Effect of the strain hardening coefficient B on the morphology of the rod flash

**Fig. 6:**
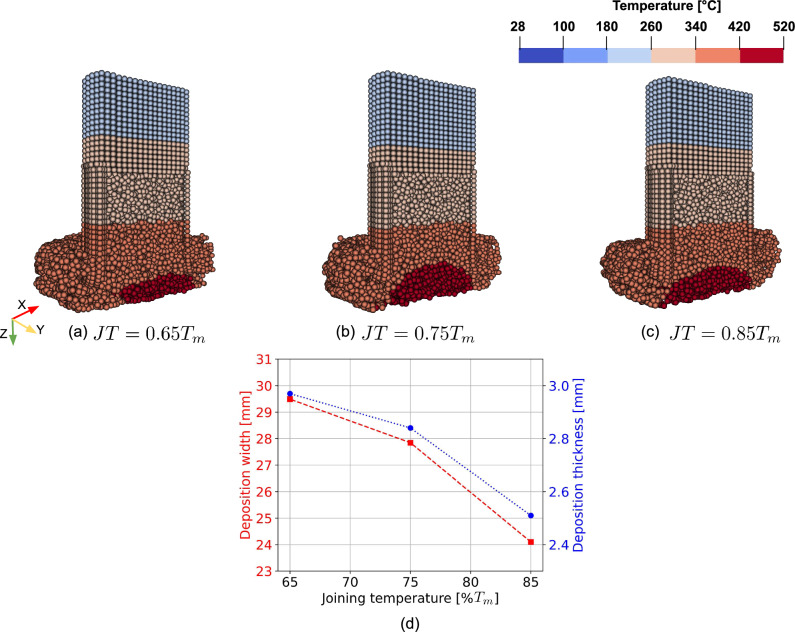
Effect of changing the joining temperature (*JT*), as described by Eq. (30), on the predicted temperature profile of the rod’s central cross section (**a**), (**b**), and (**c**), and the deposit width and thickness (**d**)

**Fig. 7:**
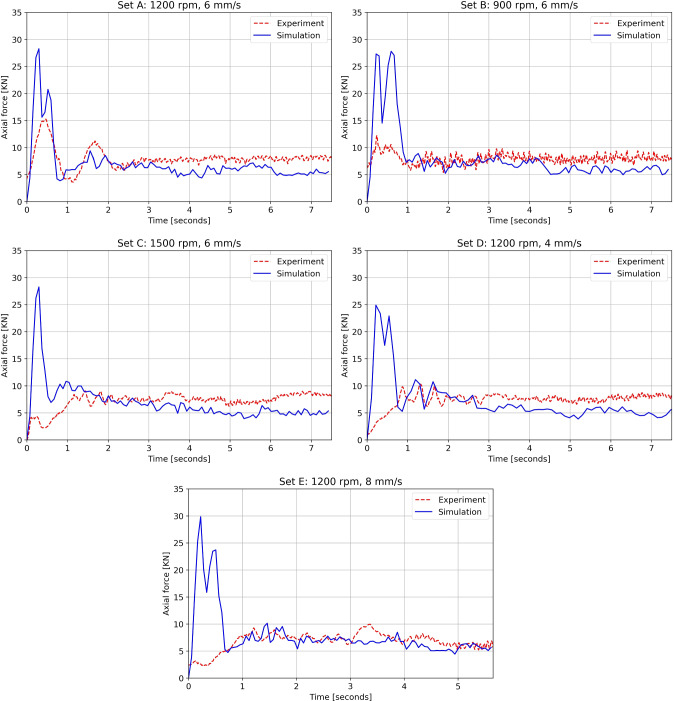
Comparison between experiment and simulation axial forces for all process parameter sets in Table 1

**Fig. 8:**
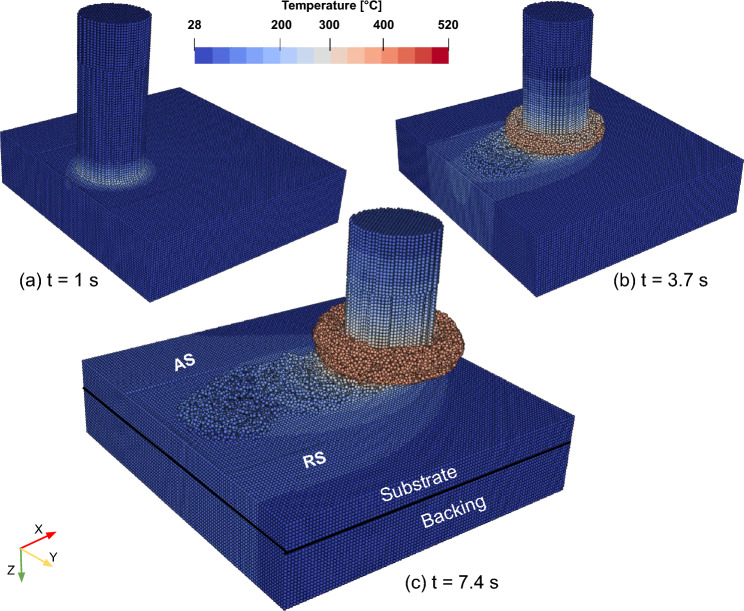
Temperature distribution during FS simulation for set B in Table 1 at **a** the end of dwelling phase, t = 1 s; **b** the middle of the simulation, t = 3.7 s; and **c** the end of the simulation, t = 7.4 s

**Fig. 9:**
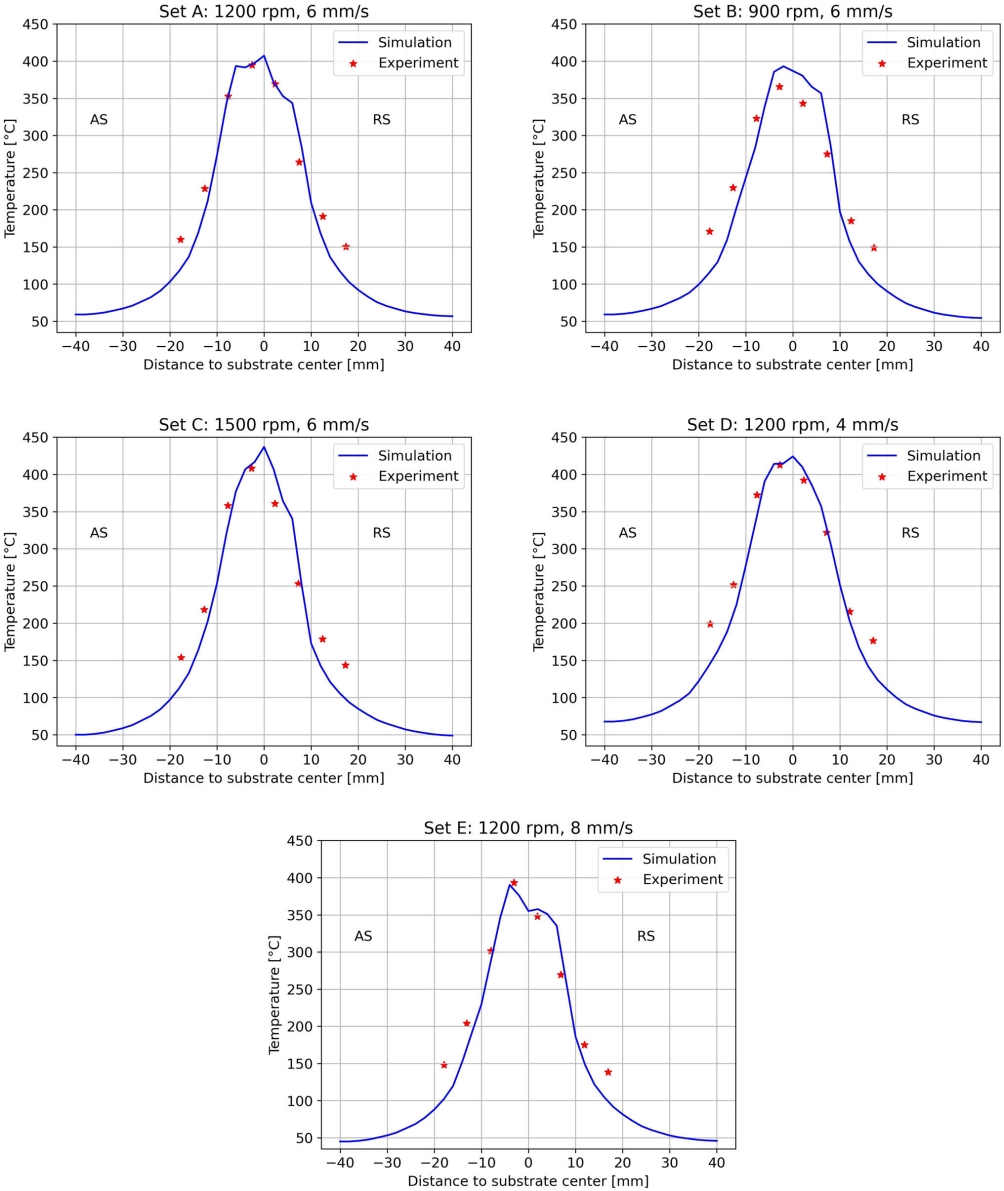
Comparison between experiment and simulation substrate maximum temperatures for all process parameter sets in Table 1

**Fig. 10:**
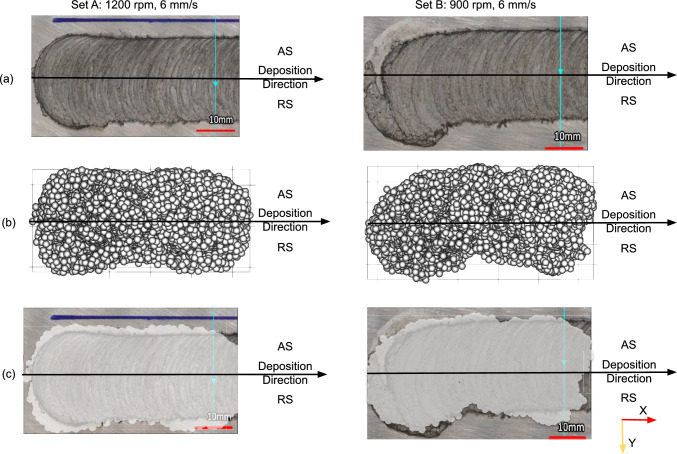
Visual comparison between experimental and simulated deposit profiles for sets A and B in Table 1: **a** experimental macrograph from the surface; **b** simulation deposit; and **c** overlapping between simulation deposit and experimental results

**Fig. 11:**
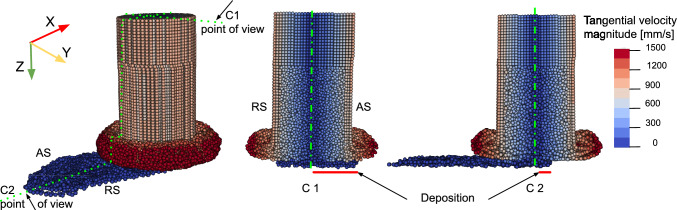
Rod cross section (C1) and longitudinal section (C2) for set B in Table 1 illustrate the deposit shifting toward the AS and forming in front of the rod’s center, respectively

**Fig. 12:**
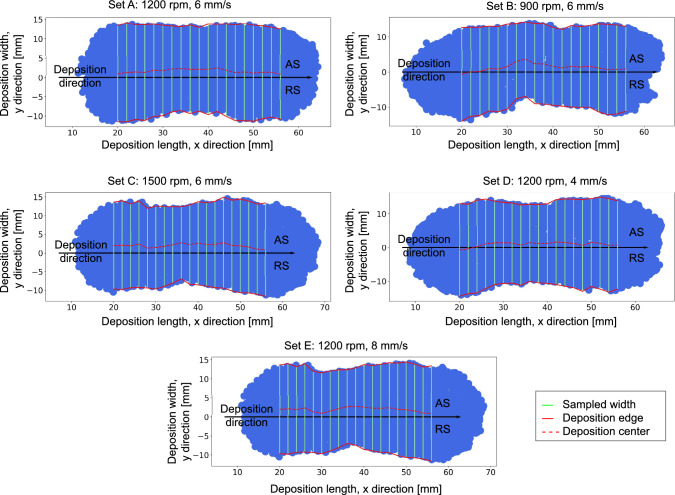
FS process simulation deposits for all process parameter sets in Table 1 with the illustration of the deposit center shifting

### Influence of model parameters

#### Influence of strain hardening of the rod material

To explore the impact of material strain hardening, i.e., the Johnson–Cook hardening parameter B of the rod material AA5083, on the FS process behavior, three simulations were performed for parameter set B in Table 1, i.e., the lowest rod rotational speed (900 rpm). The strain hardening coefficient *B* is varied between 0 and 300 MPa. It is noticed that the presence of hardening prevented the rod from achieving an ideal mushroom shape of the flash during deposition as seen for $B=0$ MPa, as illustrated in Fig. 5. Due to the irregularly formed flash, the contact area was reduced, leading also to a significantly decreased deposit width in the simulation.

At the evaluated temperatures and high strains, typically occurring during FS, the material behavior of AA5083 is significantly influenced by dynamic recrystallization (DRX) [1]. During DRX, new grains form and replace the deformed grains, which leads to a significant reduction in flow stress. This phenomenon results in a stress–strain curve, which is close to an ideal plastic behavior at elevated temperatures [50]. The simulation results, including strain hardening, indicate that the employed constitutive model struggles to capture the balance between strain hardening and thermal softening, leading to an overestimation of flow stress at high strains and elevated temperatures. Therefore, similar to the approach by Li et al. [20], strain hardening was neglected for simplicity, i.e., ideal plasticity is assumed in the following.

#### Influence of joining temperature

To assess the effect of the model parameter joining temperature on deposit geometries, simulations were also conducted for set B in Table 1 using three joining temperatures: 65%, 75%, and 85% of the rod material’s melting point. Figure 6 illustrates how varying the joining temperature influences the predicted temperature profile of the rod’s central cross section, as defined by Eq. (30), as well as the deposit width and thickness. As shown in Fig. 6, the temperature profile at the bottom of the rod became more uniform as the joining temperature increased, likely because the higher joining temperature delayed the material joining process, leading to a more consistent temperature distribution. In addition, both deposit width and thickness exhibit a decreasing trend with increasing joining temperature, see Fig. 6d, which is closer to the experimental measurements. This can be attributed to a reduced material transfer from the rod to the substrate at higher temperatures, leading to a decrease in deposited material and consequently, lower width and thickness. In the remaining simulations of this study, a joining temperature of 85% of the melting point of the rod material was chosen, which is consistent with values typically reported in the literature for FS [1].

### Axial force and temperature profile validations

The predicted axial forces for all the sets presented in this study are validated against the experimental values of the FS machine, see Fig. 7. The initial spike in the axial force is due to the perfect contact between the rod and substrate at the beginning of the simulation, where the material has not yet softened. The axial force in the simulation is slightly lower than the experimental force because the model neglects strain hardening. Overall, the axial forces are in good agreement with the experimental values and indicate that the simulation has reached steady state after the initial second.

Figure 8 illustrates the simulated temperature profiles for the rod, deposit, substrate, and backing at different time steps for set B in Table 1. At the end of the dwelling phase (Fig. 8a), heat generation is already visible at the high-shear rod–deposit contact region. As processing time increases, continuous heat generation occurs due to frictional forces and plastic deformation, which is then conducted throughout the rest of the domain, Fig. 8b. As the process advances, the substrate conducts more thermal energy to the backing, which cools down the deposit, as illustrated in Fig. 8c. Additionally, a temperature bias toward the AS is observed, highlighting the process’s inherent asymmetry, similar to the observations in the experimental and numerical studies of FS process [13, 14] as well as the SPH simulation study of the AFSD process [19].

Figure 9 compares the experimentally measured maximum substrate temperature (0.5 mm below the surface, positioned as shown in Fig. 1) with the model’s predicted maximum temperature at the same depth. This comparison was performed for all parameter sets listed in Table 1. The model can predict the maximum substrate surface temperature with a minimum error of $0.7\%$ (set E) and a maximum of $7.5\%$ (set B), which is well within the experimental scattering. Despite some variations at the substrate edges, the overall trends of the temperature profiles from experiments and simulations exhibit consistent agreement.

### Deposition geometry

To assess the agreement between the simulated and experimental deposits, simulation-deposited particles are overlaid on the experimental deposit macrographs for sets A and B in Table 1, see Fig. 10. The results show visual consistency and good agreement with experimental deposits. Moreover, the simulation successfully captured the shift of the deposit center toward the AS and the formation of the deposit in front of the rod center, as shown in Fig. 11, a phenomenon consistently observed in experimental studies [10, 51]. The deposit center shifting for all processing parameters listed in Table 1 is quantitatively illustrated in Fig. 12.

**Fig. 13:**
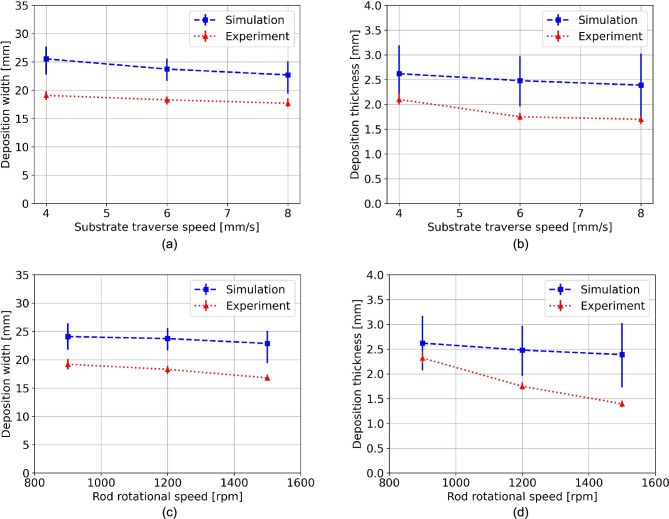
Comparison between experimental and simulation deposit width and thickness for variation of traverse speeds (**a**) and (**b**), for variation of rotational speeds (**c**) and (**d**), respectively

Figure 13 shows the comparison of the deposited material width and thickness with experimental results for all process parameter sets in Table 1. Although the simulation results show quantitatively a slight offset, the simulation successfully replicated the observed experimental trends: Deposit width and thickness decreased as traverse or rotational speeds increased. One source for deviations might be attributed to the discretization size. Experimentally, by increasing one of the speeds, the deposit becomes thinner and narrower. This makes it more difficult for the model to predict the new fine geometries at the same selected particle size. Decreasing the particle size might help reduce the deviation; however, it will increase the running time polynomially.

### Material flow pattern

**Fig. 14:**
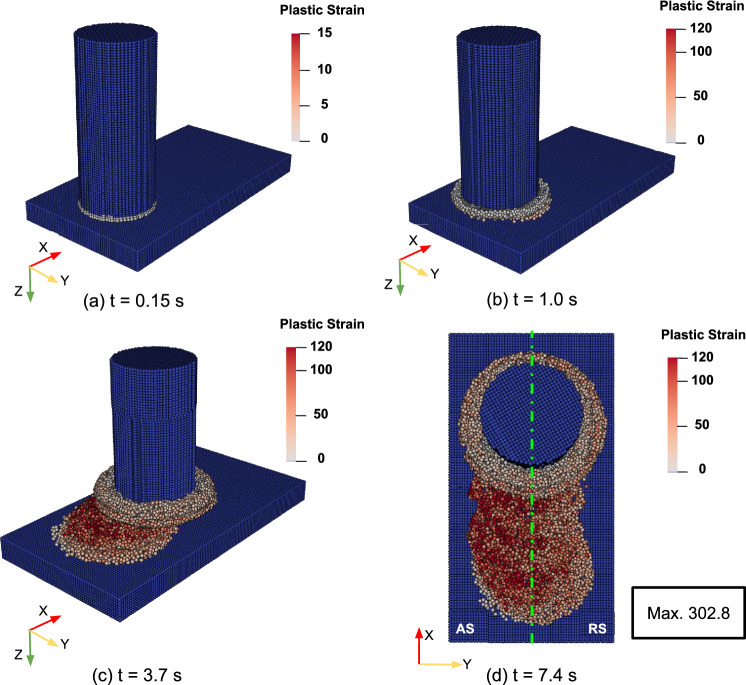
Snapshots of the effective plastic strain during FS simulation for set B in Table 1 at **a** process start, t $=$ 0.15 s; **b** end of the dwelling phase, t $=$ 1.0 s; **c** middle of the simulation, t $=$ 3.7 s; and **d** end of the simulation, *t* = 7.4 s, (top view)

Figure 14 displays snapshots of the process simulation for set B in Table 1, showing the effective plastic strain at different time steps. The results illustrate that the rotating rod starts by rubbing the substrate and initially deforms its lower part as shown in Fig. 14a. At the end of the dwelling phase, the deposit starts to form, see Fig. 14b. The process then moves into the deposition phase, where the rotating rod is progressively pushed against the developing deposit while the substrate moves at a constant traverse speed. The compressive feeding forces and high shear forces from the rod deform the deposit and gradually form the rod flash, see Fig. 14c. The high plastic strain primarily develops over the rod flash and deposit, with higher values on the AS compared with the RS as shown in Fig. 14d. This finding was also observed in FEM simulation studies of the FS process [13, 14] and aligns with experimental data [52], which observed finer grains on the AS than on the RS, indicating a higher strain on the AS.

**Fig. 15:**
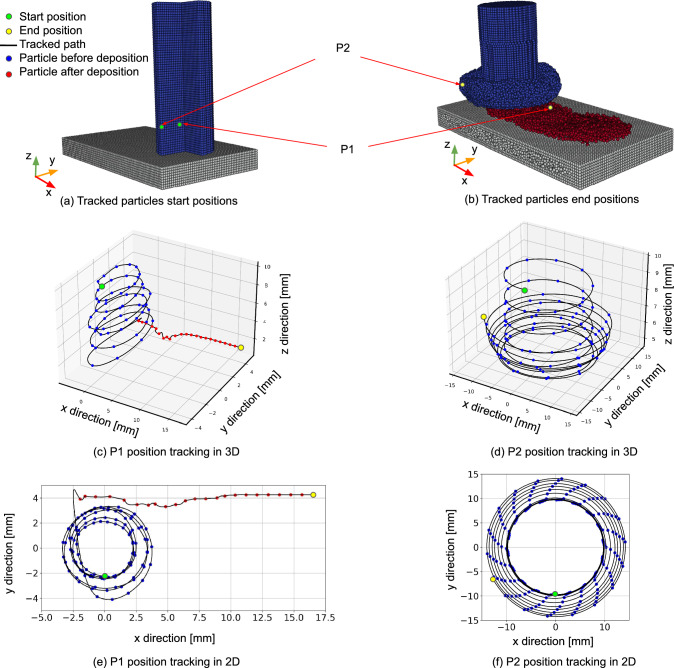
Position tracking for two particles, P1 and P2, at radii of 2 mm and 10 mm, measured 10 mm above the rod–substrate interface: **a** initial positions of P1 and P2; **b** final positions of P1 and P2; **c** and **d** 3D position tracking of P1 and P2, respectively; and **e** and **f** 2D position tracking of P1 and P2, respectively. The results correspond to set B in Table 1

The material flow pattern is explicitly monitored by tracking two particles, P1 and P2, one located in the inner part of the rod (at a radius of 2 mm) and one on its outer surface, respectively, positioned 10 mm below the initial rod–substrate contact surface as illustrated in Fig. 15. The coordinates in these plots reflect the actual locations of the particles within the rod or on the deposited layer, in case deposition occurred. For P1, see Fig. 15c and e, the particle follows a helical trajectory, moving downward to the substrate surface before leaving the rod and becoming part of the deposited layer. During the early stages of deposition, the particle’s path is irregular, indicating interactions with rod particles in contact. For P2, the particle exhibits two distinct helical trajectories, see Fig. 15d: At first, the particle is descending while it remains in the rod. At a later stage, where the particle is ascending, it transitions into the flash leading to the increasing size of the flash during the process (Fig. 15f).

**Fig. 16:**
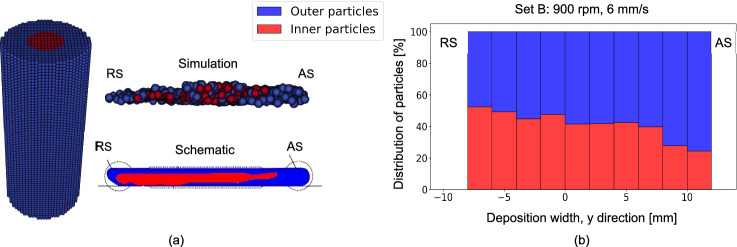
Distribution of rod particles within the deposit: **a** initial rod particles, with inner particles shown in red and outer particles in blue, along with a cross-sectional view at the deposit midpoint illustrating inner and outer particles, and a schematic prediction of particle distribution based on experimental findings [51] and **b** the distribution of inner and outer particles across the deposit width along its length. These results correspond to set B in Table 1

The simulation effectively captures the material deposition behavior during processing, which aligns with experimental observations [51]. Specifically, the inner material of the rod is deposited toward the RS, while the outer material shifts toward the AS, as illustrated in Fig. 16. To visualize this behavior, initial rod particles within an 8 mm diameter are marked and tracked throughout the deposition process. The simulated deposit accurately reflects the schematic pattern observed experimentally for special consumable studs containing two different aluminum alloys [51] (Fig. 16a). Furthermore, an analysis of the distribution of inner and outer particles across the deposit width (Fig. 16b) shows a pronounced shift of inner particles toward the RS, consistent with experimental observations [51]. The simulation suggests that this phenomenon is due to the higher frictional force on the AS compared to the RS, resulting from a higher relative velocity, as described by Eq. (15). Consequently, the outer surface particles tend to deposit more on the AS, pushing the early deposited inner particles toward the RS. For the distribution of the rod outer particles in the deposit and formed flash, see “Appendix F”.

**Fig. 17:**
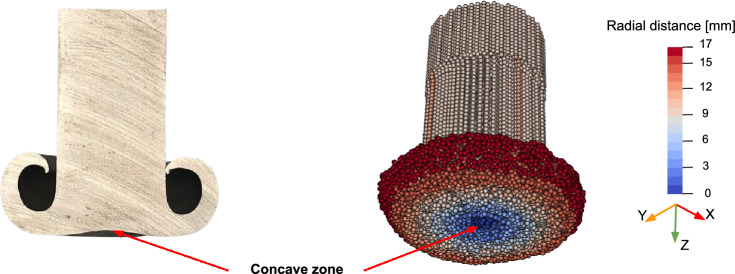
Comparison between predicted and experimental rod shapes after deposition, illustrating the model’s ability to predict the experimentally observed concave profile of the rod

Finally, the model accurately predicted the concave shape at the deposition tip within the consumable rod, as observed experimentally [1], see Fig. 17. This concave shape occurs because the rod’s temperature is highest at the center, which accelerates material joining by quickly satisfying the temperature condition in the joining criterion. Furthermore, the shear stress condition is quickly satisfied at the rod center because the allowable shear stress decreases as temperature increases, which facilitates faster material joining.

Overall the simulation effectively captures the experimental characteristics. While there are some small deviations, this is expected due to the inherent challenges of modeling the highly nonlinear process. Nonetheless, the close agreement between the simulation and experimental results underscores the robustness of the presented simulation approach.

The developed solver will be released as open-source software, encouraging further development and collaboration within the research community. Future work will focus on using the adaptive particle refinement method, enhancing the model’s capabilities to handle finer particles, and upgrading the solver to run on multi-GPUs.

## Conclusion

A GPU-accelerated, meshfree SPH model was developed and validated for simulating and understanding the FS process. The model represents rod and substrate materials as thermo-elastic viscoplastic solids using the JC material model, with the backing plate modeled as a rigid body. Simulation efficiency was enhanced through particle switching techniques and sub-domain division, while stability was ensured using Monaghan’s artificial viscosity, artificial stress, and CSPM.

The SPH model was validated against experimental data, demonstrating excellent agreement in terms of axial force, temperature profiles, and deposit geometries for a wide range of process parameters. Additionally, the model is employed to investigate and understand the deposition mechanisms during FS by analyzing the material flow, deposited material distribution, and rod flash formation. The findings can be summarized as follows:

- Overestimated material hardening introduces irregularities in the rod flash, while increasing the joining temperature improves temperature uniformity in the rod and reduces the thickness and width of the deposit.
- Higher rod rotational and substrate traverse speeds reduce the thickness and width of the deposit, whereas lower traverse speeds increase maximum substrate temperatures.
- Process asymmetry is observed with deposition shifted and temperature bias toward the AS.
- High plastic strain is concentrated in the rod flash and deposit, with higher strain on the AS compared to the RS.
- The inner material of the consumable rod follows a helical trajectory, descending to the deposit surface and deforming further post-deposition due to interactions with the rod material.
- The outer surface of the consumable rod exhibits dual helical trajectories: one descending within the rod and another ascending, contributing to flash formation.
- The deposition tip of the rod develops a concave shape, driven by elevated temperatures at its center.

The validated SPH model serves as a robust tool for analyzing the effects of process parameters, including material properties, geometries, rod rotational speed, feed rate, and substrate traverse speed, on deposition field variables such as temperature, strain, and deposit geometries, allowing a deeper insight into the complex material flow mechanisms during FS.

## Figures and Tables

**Fig. 18:**
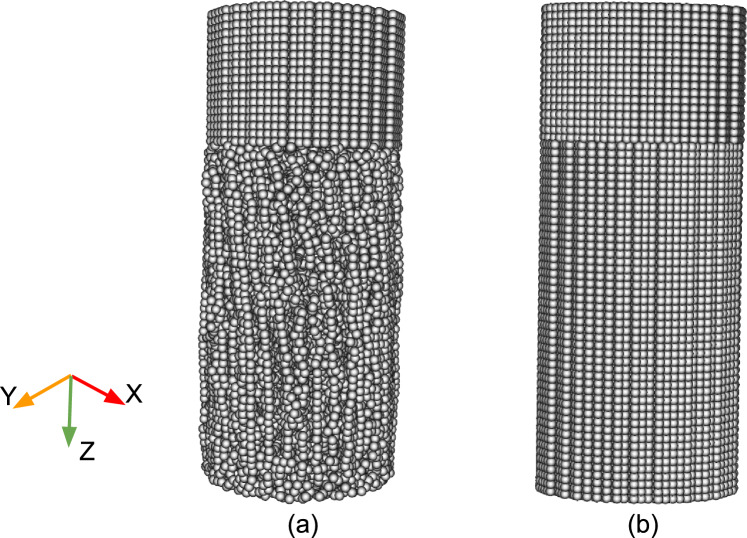
Effect of using CSPM on the rod outer surface particles at 0.2 s of simulation: **a** shows the onset of irregular distribution without CSPM and **b** demonstrates the preserved uniformity when CSPM is applied

**Fig. 19:**
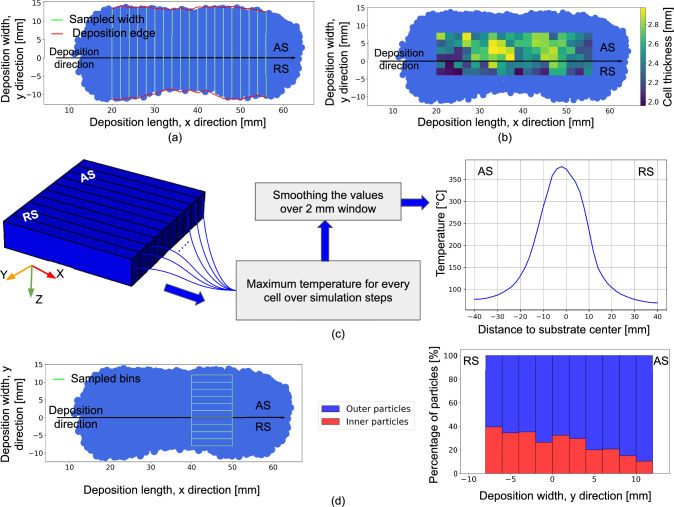
Post-processing operations: **a** deposit sampling for width measurement; **b** deposit sampling for thickness measurement; **c** schematic illustrating maximum substrate temperature sampling and data extraction; and **d** calculation of the percentage distribution of the rod inner and outer particles across the deposit width

**Fig. 20:**
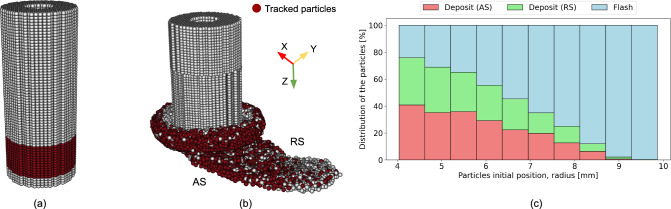
Tracking of particles in the outer region of the rod (dark red), defined by $\text {diameter} > 8$ mm, 3.75 mm above the initial rod–substrate contact surface, and over a length of 10.5 mm, throughout the simulation of FS process **a** tracked particles initial positions; **b** tracked particles final positions; and **c** distribution of the tracked particles ending up in the flash and the deposit (AS and RS) to their initial positions. These results correspond to set B in Table 1

## Data Availability

The solver is released as open-source code via GitHub (https://github.com/SPH-SSMP/FS-SPH-GPU) and the simulation results are online available at Zenodo (10.5281/zenodo.16639548).

## Notation

The notation used throughout this paper is as follows: *x* denotes a scalar, where ${\varvec{x}}$ represents a vector and ${\varvec{X}}$ a second-order tensor, i.e., a matrix. Exceptions are clearly noted in the following, such as the stress ${\varvec{\sigma }}$ and strain tensor ${\varvec{\varepsilon }}$. ${\varvec{x}}_1\cdot {\varvec{x}}_2$ denotes the dot product of two vectors, where ${\varvec{x}}_1\circ {\varvec{x}}_2$ is the Hadamard product and ${\varvec{x}}\otimes {\varvec{x}}$ represents the dyadic product of two vectors. ${\varvec{X}}\,{\varvec{x}}$ describes the typical matrix–vector product and ${\varvec{X}}:{\varvec{X}}$ is the double contraction of the tensor ${\varvec{X}}$. ${\varvec{\nabla }}$ is the gradient operator, $\textrm{div}({\varvec{x}})$ represents the divergence operator, i.e., $\textrm{div}(x)={\varvec{\nabla }}\cdot {\varvec{x}}$ and ${\dot{x}}$ represents the time derivative of *x*. The L2-norm of a vector is denoted as $||{\varvec{x}}||$.

## Stabilizers

The artificial viscosity $\Pi _{ij}$ in Eq. (2) is used to reduce the unphysical oscillations in the numerical results. In this work, the Monaghan-type artificial viscosity [45] is used, which can be written as

B.1 $$\begin{aligned} \Pi _{ij}= {\left\{ \begin{array}{ll}\frac{-\alpha _{\Pi } {c}_{i j} \emptyset _{i j}+\beta _{\Pi } \emptyset _{i j}^{2}}{{\rho }_{i j}}, &  {\varvec{v}}_{i j} \cdot {\varvec{x}}_{ij} <0 \\ 0, &  {\varvec{v}}_{i j} \cdot {\varvec{x}}_{i j} \ge 0\end{array}\right. } \end{aligned}$$

B.2 $$\begin{aligned} \text {with} \quad \emptyset _{i j} =\frac{h_{i j} {\varvec{v}}_{i j} \cdot {\varvec{x}}_{i j}}{\left\| {\varvec{x}}_{i j}\right\| ^{2}+\varphi }, \end{aligned}$$

where $\rho _{i j}$, $h_{i j}$, and ${c}_{i j}$ represent the average of densities, smoothing lengths, and speeds of propagating sound wave for particles i and j, respectively. $\alpha _{\Pi }$ and $\beta _{\Pi }$ are constants. The constant $\varphi$ is set to 0.01 $h_{i j}^{2}$ to prevent two particles from approaching each other, leading to numerical issues. A recent study on the effects of these parameters and their choices is provided by Mao et al. [53].

The artificial stress ${\varvec{R}}_{ij}$ is introduced in Eq. (2) to address the issue of tensile instability, as discussed by Monaghan et al. [46]. To determine the stress tensor ${\varvec{R}}_{i}$ for a given particle *i*, the principal stresses of the stress state $\varvec{\sigma }_i$ must be analyzed. This involves performing an eigenvalue decomposition of the stress tensor $\varvec{\sigma }_i$, which can be expressed as:

B.3 $$\begin{aligned} \varvec{\sigma }_i={\varvec{Q}}_i \cdot \varvec{\Lambda }_i \cdot {\varvec{Q}}_i^{-1}={\varvec{Q}}_i \cdot \varvec{\Lambda }_i \cdot {\varvec{Q}}_i^{T}, \end{aligned}$$

where ${\varvec{Q}}_i^{-1}={\varvec{Q}}_i^{T}$ since $\varvec{\sigma }_i$ is symmetric. The principal stresses ${\Lambda }_{i,\xi \xi }$, which are the eigenvalues in the diagonal matrix $\varvec{\Lambda }_i$, are modified to form the matrix $\varvec{\Lambda }_i^{\prime }$. This modification involves multiplying each positive (tensile) stress component by a user-defined intensity parameter $\epsilon$. The elements ${\Lambda }_{i,\xi \xi }^{\prime }$ in the modified matrix $\varvec{\Lambda }_i^{\prime }$ are given by

B.4 $$\begin{aligned} {\Lambda }_{i,\xi \xi }^{\prime } = \left\{ \begin{array}{ll} -\epsilon {\Lambda }_{i,\xi \xi }, &  {\Lambda }_{i,\xi \xi }>0 \\ 0, &  {\Lambda }_{i,\xi \xi }\le 0 \end{array} \right. \end{aligned}$$

with ${\xi } \in [x,y,z]$. These modified eigenvalues are used to compute ${\varvec{R}}_{i}$ by rotating them back to their original frame

B.5 $$\begin{aligned} {\varvec{R}}_{i}= {\varvec{Q}}_i^{T} \cdot \varvec{\Lambda }_i^{\prime }\cdot {\varvec{Q}}_i. \end{aligned}$$

${\varvec{R}}_{ij}$ can be found by

B.6 $$\begin{aligned} {\varvec{R}}_{i j}=\left[ {\varvec{R}}_{i}+{\varvec{R}}_{j}\right] \ \left[ \frac{W_{i j}}{W(\Delta x)} \right] ^{\gamma }, \end{aligned}$$

where $W(\Delta x)$ is the smoothed kernel value at the initial particle spacing $\Delta x$, which can be obtained by replacing $\Vert {\varvec{x}}_{i j}\Vert$ in Eq. (5) by $\Delta x$. $\gamma$ is another user parameter that causes the influence of neighboring artificial stresses to decline.

## CSPM

Excluding CSPM from the simulation causes uneven particle distribution on the rod’s outer surface. Without applying CSPM, particles at the boundary have less kernel support, leading to clustering and void problems (Fig. 18). CSPM fixes this by adjusting the kernel gradient and keeping particle spacing uniform.

## Average normal vector calculations $\textit{n}_{ij}^{av}$

The FS process includes interaction between two deformable bodies: the rod and substrate. To account for this interaction, a free surface detection algorithm similar to the one used by Stubblefield et al. [18] has been employed. This algorithm first calculates the center of mass ${\varvec{x}}_{i}^{\textrm{com}}$ for each particle neighborhood as follows

D.1 $$\begin{aligned} {\varvec{x}}_{i}^{\textrm{com}}=\frac{1}{M} \sum _{j=1, \textrm{Part}_{j} = \textrm{Part}_{i}}^{N_{j}} m_{j} {{\varvec{{x}}}_{i j}} . \end{aligned}$$

The statement $\textrm{Part}_{j} = \textrm{Part}_{i}$ ensures that the *i*th and *j*th particles belong to the same body. ${N_{j}}$ represents the total number of particles in the neighboring cluster within the smoothing length *h*, and *M* is their total mass. Then, it selects candidate surface particles based on a threshold value that represents how much the center of mass of the particle neighborhood ${\varvec{x}}_{i}^{\textrm{com}}$ is shifted. The list of surface particles $S_{\textrm{list}}$ is determined as follows

D.2 $$\begin{aligned} S_{\textrm{list}} = \left\{ \begin{array}{ll} \text {selected}, &  \Vert {\varvec{x}}_{i}^{\textrm{com}}\Vert \ge 0.05 h \\ \text {not selected}, &  \text {otherwise}. \end{array} \right. \end{aligned}$$

Finally, for each particle in $S_{\textrm{list}}$, a conical region is scanned around the particle, aligned with the direction of the particle normal $\frac{{\varvec{x}}_{i}^{\text {com}}}{\Vert {{\varvec{x}}_{i}^{\text {com}}\Vert }}$. If another particle from the same body is found within this conical region, the original particle is removed from $S_{\textrm{list}}$. This revision clarifies that the conical region is aligned with the direction of the contact force. The scanning procedure is similar to the one employed by Marrone et al. [54]. This approach helps to ensure accurate surface detection while avoiding overly restrictive filtering.

To determine ${\varvec{{n}}}_{ij}^{av}$, the smoothed vector of the surface particles ${\varvec{{n}}}_{i}^{s}$ is calculated first by taking the average of all the surface normals of the neighboring particles ${\varvec{{n}}}_j$ that are also in the surface list $S_{list}$. Numerically, ${\varvec{{n}}}_j$ and ${\varvec{{n}}}_{i}^{s}$ can be expressed as

D.3 $$\begin{aligned} {\varvec{{n}}}_j=\left\{ \begin{array}{lc}\frac{{\varvec{{x}}}_{j}^{\text {com}}}{\left\| {\varvec{{x}}}_{j}^{\text {com}}\right\| }, & \text {Surface}_\text {list}=1 \\ 0, & \text {Otherwise}\end{array}\right. \end{aligned}$$

D.4 $$\begin{aligned} {\varvec{{n}}}_{i}^{s}=\frac{1}{N_{s}} \sum _{j=1}^{N_{s}} {\varvec{{n}}}_j, \end{aligned}$$

where $N_s$ is the number of neighboring surface particles including the particle *i*. The average vector between two particles ${{\varvec{n}}}_{ij}^{av}$ is then calculated based on the angle $|\theta _{ij}|$ between their smoothed vectors, ${{\varvec{n}}}_{i}^{s}$ and ${{\varvec{n}}}_{j}^{s}$, as well as a user-defined angle $\theta _{c}$. The average vector is determined as follows:

- First, compute the angle $|\theta _{ij}|$ between the two smoothed vectors
  $$\begin{aligned} |\theta _{ij}|=\angle \left( -{\textbf {{n}}}_{i}^s,{{\varvec{{n}}}}_{j}^s\right) , \end{aligned}$$
- $\textrm{if}(|\theta _{ij}| < \theta _{c})$, evaluate the following expression
  $$\begin{aligned} \max \left( {\varvec{{x}}}_{ij} \cdot {\varvec{{n}}}_{i}^s, - {\varvec{{x}}}_{ij} \cdot {\varvec{{n}}}_{j}^s, {\varvec{{x}}}_{ij} \cdot \frac{ {\varvec{{n}}}_{i}^s - {\varvec{{n}}}_{j}^s}{\left\| {\varvec{{n}}}_{i}^s - {{\varvec{{n}}}}_{j}^s\right\| }\right) , \end{aligned}$$

$\circ$: $\textrm{if}({\varvec{{x}}}_{ij} \cdot {\varvec{{n}}}_{i}^s)$ is the maximum, then ${\varvec{{n}}}_{ij}^{av} = {\varvec{{n}}}_{i}^s$.
$\circ$: $\textrm{if}(-{\varvec{{x}}}_{ij} \cdot {\varvec{{n}}}_{j}^s)$ is the maximum, then ${\varvec{{n}}}_{ij}^{av} = -{\varvec{{n}}}_{j}^s$.
$\circ$: $\textrm{if}({\varvec{{x}}}_{ij} \cdot \frac{{\varvec{{n}}}_{i}^s - {\varvec{{n}}}_{j}^s}{\left\| {\varvec{{n}}}_{i}^s - {\varvec{{n}}}_{j}^s \right\| })$ is the maximum, then ${\varvec{{n}}}_{ij}^{av} = \frac{{\varvec{{n}}}_{i}^s - {\varvec{{n}}}_{j}^s}{\left\| {\varvec{{n}}}_{i}^s - {\varvec{{n}}}_{j}^s \right\| }$.

- $$\textrm{if}(|\theta _{ij}| > \theta _{c})$$, then
  D.5 $$\begin{aligned} {\varvec{{n}}}_{ij}^{av}=\frac{{\varvec{{n}}}_{i}^s-{\varvec{{n}}}_{j}^s}{\left\| {\varvec{{n}}}_{i}^s-{\varvec{{n}}}_{j}^s\right\| }. \end{aligned}$$

## Post-processing of simulation results

The deposited particles are projected onto a 2D plane and then sampled into 19 steps to calculate the deposit width from the simulation results. The maximum and minimum particle positions in the *y* direction are computed at each step to form the outer edges. To avoid the influence of the dwelling and ending phases, the measurements were taken from 20 to 55 mm along the deposit length, as shown in Fig. 19a. The same method is extended to calculate the thickness by sampling the deposited material volume into 30 cells covering an area of $12 \times 35 \ \textrm{mm}^{2}$ in the middle of the deposit to avoid the side edges. The thickness is measured by calculating the maximum height in every cell as illustrated in Fig. 19b. To capture the continuous maximum surface temperature from the discrete model data, the substrate width was divided into 2 mm cells extending from 0.5 mm below the surface and covering the entire length. For each simulation step, the maximum temperature within each cell was recorded, and the highest temperature across all steps was selected for each cell. A smoothed curve was then generated by averaging these maximum temperatures over a 2 mm window, see Fig. 19c for a schematic overview. To compare the distribution of the inner and outer particles of the rod within the deposit, the inner particles are identified within a specified radius and tracked throughout the deposition process. A stacked bar plot is used to illustrate the proportions of inner and outer particles within 2 mm bins along the deposit width. For each bin, the percentage amounts of inner and outer particles are displayed for a deposit length of 10 mm, as illustrated in Fig. 19d.

## Outer rod particle distribution in the deposit and flash

Particles located in the outer region of the rod–specifically, those at a diameter greater than 8 mm, 3.75 mm above the initial rod–substrate contact surface, and over a length of 10.5 mm–have been tracked throughout the deposit and flash (Fig. 20). It was found that 68% of the particles in the outer region of the rod ended up in the flash, while the remaining particles were deposited. Of those deposited, 67% were found on the AS. Figure 20c maps the percentage of particles in the flash and deposit (AS and RS) based on their initial positions. As particles approach the rod’s outer surface, more particles end up in the flash than in the deposit, while the ratio of particles deposited on the AS to those on the RS remains nearly constant. These observations are consistent with a recent experimental study [51].

## Footnotes

1 Plastic strain condition is introduced to the joining criterion to avoid spurious numerical artifacts.
